# Giant seminoma of the testis (2.51 kg) in a setting of severe occupational and lifestyle exposures: a case report and pathophysiological insights

**DOI:** 10.3389/fmed.2026.1850712

**Published:** 2026-05-28

**Authors:** Hong-Ping Tao, Bin Chen, Ya-Ran Chen, Geng Yang, Kai-Li Mao

**Affiliations:** 1 Department of Urology, The First People's Hospital of Wuyi County, Jinhua, China; 2 Department of Clinical Medicine, School of Medicine, Hangzhou City University, Hangzhou, China; 3 Department of Pharmacy, The Quzhou Affiliated Hospital of Wenzhou Medical University, Quzhou People’s Hospital, Quzhou, China

**Keywords:** case report, environmental exposure, giant seminoma, lifestyle, testicular germ cell tumors

## Abstract

We report a case of a giant testicular seminoma in a late 30s male with a two-year history of progressive left scrotal enlargement, who presented with a 22 × 14 × 12 cm mass weighing 2.51 kg. The patient had a significant occupational history of working as a barbecue vendor with daily exposure to high-temperature cooking oil fumes for 3 years, accompanied by extreme lifestyle disruption including approximately 20 working hours per day, less than 4 h of sleep, and heavy smoking and alcohol consumption. Although the exact timing of tumor initiation cannot be determined, these exposures may have contributed to tumor progression in a malignancy that likely had a prolonged preclinical phase. All serum tumor markers, including AFP, hCG, and LDH, were within normal limits. Radical orchiectomy was performed, and histopathology combined with immunohistochemistry (PLAP+, CD117+, SALL4+, D2-40+, CKpan−, CD30−) confirmed pure seminoma. This case highlights that in addition to classical risk factors such as cryptorchidism, chronic environmental exposure to carcinogens (e.g., polycyclic aromatic hydrocarbons from cooking fumes) and severe chronic sleep deprivation may represent potentially modifiable factors that warrant further investigation into their possible association with testicular germ cell tumors. Delayed diagnosis due to occupational neglect allowed the tumor to reach an exceptional size. Although causal associations remain to be established, preventive strategies that reduce exposure to environmental carcinogens, address occupational stress, ensure adequate sleep, and promote routine scrotal self-examination and timely medical consultation may be considered on general health grounds.

## 1 Introduction

Testicular germ cell tumors (TGCTs) are the most common malignancies in young and middle-aged men, accounting for approximately 1 to 1.5% of all male cancers and 5% of urological tumors worldwide (1). Although the global incidence remains relatively low (4–8 per 100,000 males annually), a steady increase has been observed in several developed countries over recent decades (2). Cryptorchidism remains the most well-established risk factor, particularly for seminoma (3). Other recognized factors include family history and cryptorchism (4). However, these factors alone do not fully explain the rising incidence, suggesting the involvement of environmental and lifestyle-related exposures.

Emerging evidence has implicated endocrine-disrupting chemicals (EDCs), such as organochlorine pesticides and phthalates, in the pathogenesis of TGCTs through the testicular dysgenesis syndrome (TDS) hypothesis (5). Furthermore, occupational and dietary exposure to polycyclic aromatic hydrocarbons (PAHs), commonly found in cooking fumes, grilled meats, and tobacco smoke, has been associated with increased cancer risk (6, 7). Despite these insights, the role of chronic, real-world exposure to cooking oil fumes combined with severe lifestyle disruption (e.g., chronic sleep deprivation, high stress) remains poorly characterized in the urological literature.

Delayed diagnosis is another critical but often overlooked issue in TGCTs, particularly in low-resource settings or among individuals with heavy occupational workloads. Giant seminomas (e.g., >10 cm or >1 kg) are exceptionally rare in modern clinical practice. Such cases provide a unique opportunity to explore the cumulative impact of environmental, behavioral, and socioeconomic factors on tumor progression.

Here, we report a case of a giant seminoma weighing 2.51 kg in a late 30s male with a two-year history of progressive scrotal enlargement, who had sustained chronic exposure to high-temperature cooking fumes and extreme sleep deprivation (≤4 h/day) for 3 years preceding admission. Notably, the patient also had a long-standing history of heavy smoking, which may have contributed to an earlier initiation of carcinogenesis. We do not imply a direct short-term causal link between the three-year occupational exposure and tumor initiation; rather, these factors may have accelerated tumor growth or promoted progression in a neoplasm with an indolent preclinical phase. This case aims to describe the temporal co-occurrence of modifiable environmental and lifestyle factors with TGCT development, generating hypotheses for future epidemiological studies, and to emphasize the importance of early diagnosis and preventive strategies.

## 2 Case presentation

### 2.1 History of present illness

A late 30s male, self-employed as a barbecue vendor, was admitted to our hospital in February 2026, with a chief complaint of a left scrotal mass that had been present for 2 years and had rapidly enlarged over the past 5 months. The patient first noticed a slight enlargement of the left scrotum 2 years prior, with occasional pain managed by self-medication with ibuprofen. Over the following 2 years, the mass gradually enlarged. Five months before admission, it reached approximately the size of an egg and began to grow rapidly, accompanied by dull pain and a dragging sensation. He also reported significant weight loss (13 kg) and declining physical strength. Three years prior, he had started a barbecue business in Wuyi County, working long hours in a smoky environment. He worked approximately 20 h daily, with less than 4 h of rest per day. He consumed approximately 1,500 mL of beer (3% alcohol) and smoked 20 cigarettes daily.

### 2.2 Physical examination

**Figure 1:**
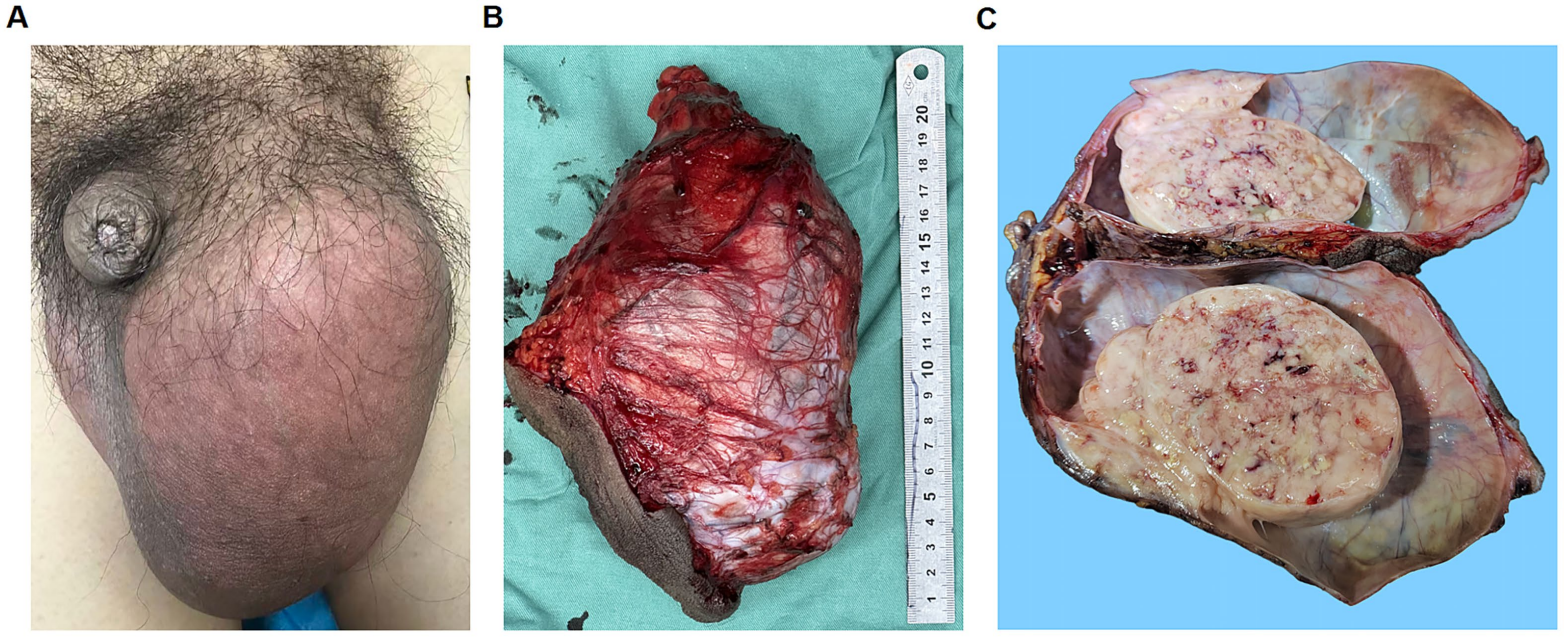
Macroscopic features of the scrotal mass. **(A)** Clinical photograph showing the scrotal mass *in situ* (anterior view). **(B)** Gross appearance of the resected specimen. **(C)** Cut surface of the mass revealing its internal architecture.

Vital signs: pulse 87 bpm, blood pressure 110/65 mmHg, temperature 36.6 °C, respiratory rate 18/min. Abdominal examination was unremarkable. A left scrotal mass measuring approximately 22 × 14 × 12 cm was palpable (Figure 1); it was irregular, firm, poorly mobile, and non-reducible. Transillumination was negative. The left testis and epididymis were not discernible. The right testis was normal in size and consistency.

### 2.3 Laboratory findings

All measured tumor markers and hormone levels, including prolactin (215.5 μIU/mL), FSH (10.2 mIU/mL), LH (5.1 mIU/mL), testosterone (20.54 nmol/L), estradiol (139.3 pmol/L), hCG (5.04 mIU/mL), LDH (1,216 U/L), ferritin (553.90 ng/mL), AFP (1.98 ng/mL), CEA (1.21 ng/mL), CA-125 (6.4 KU/L), CA 19–9 (3.41 kU/L), and tPSA (0.58 ng/mL), were within normal reference ranges.

### 2.4 Imaging

**Figure 2:**
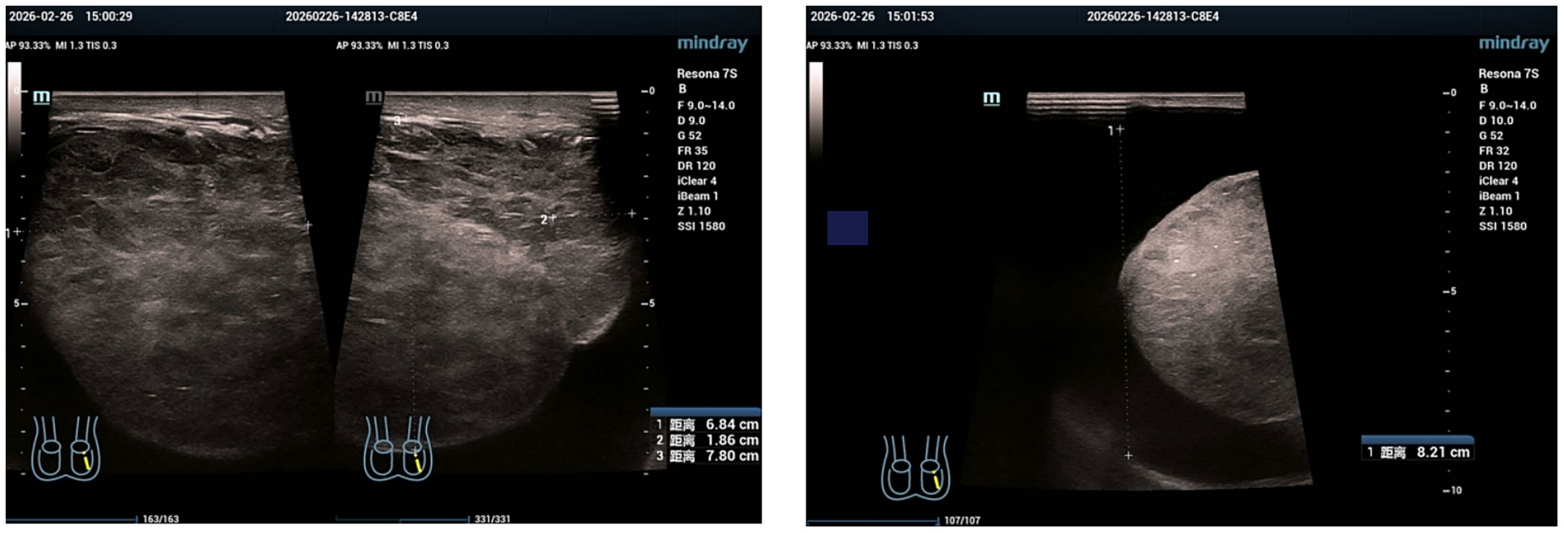
Abdominal ultrasound examination of the testicles and epididymis. The left testicle and epididymis have enlarged volume with altered echoes, there is a mass in the testicle and epididymis, there is effusion in the left testicular tunica albuginea cavity, and there is microcalcification in the right testicle.

**Figure 3:**
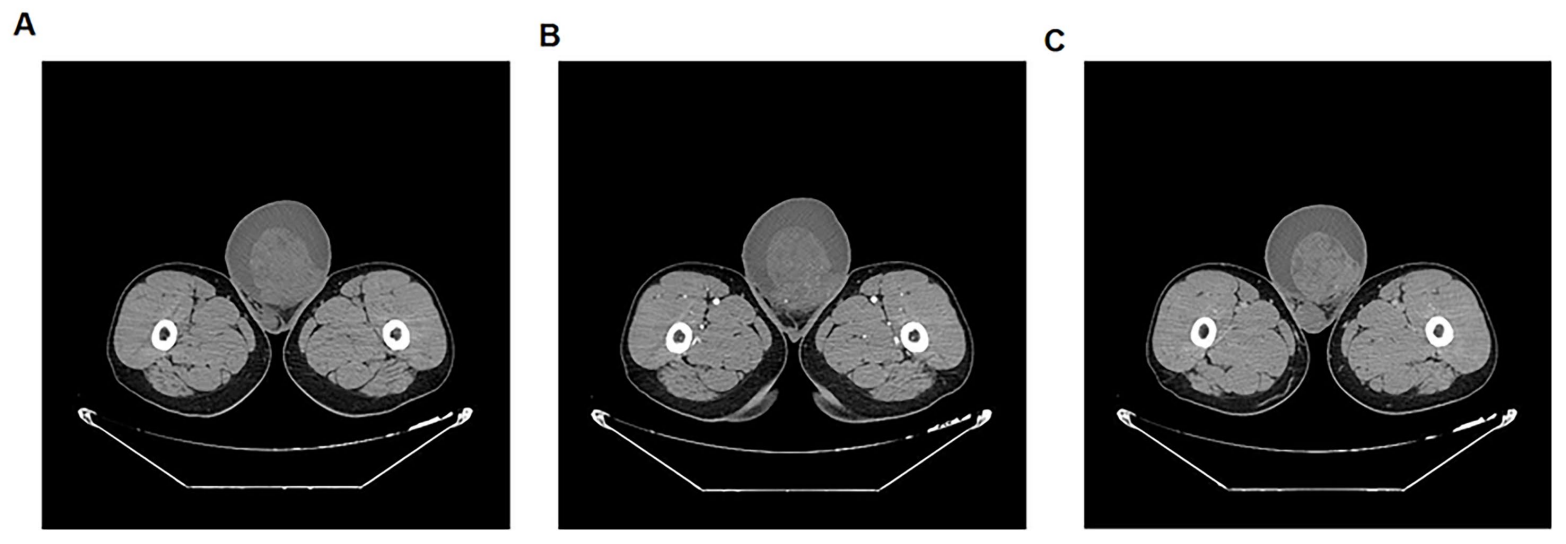
The conventional plain scan (without contrast agent injection) **(A)**, arterial phase **(B)**, and venous phase **(C)** images of the testicular enhanced CT examination.

**Figure 4:**
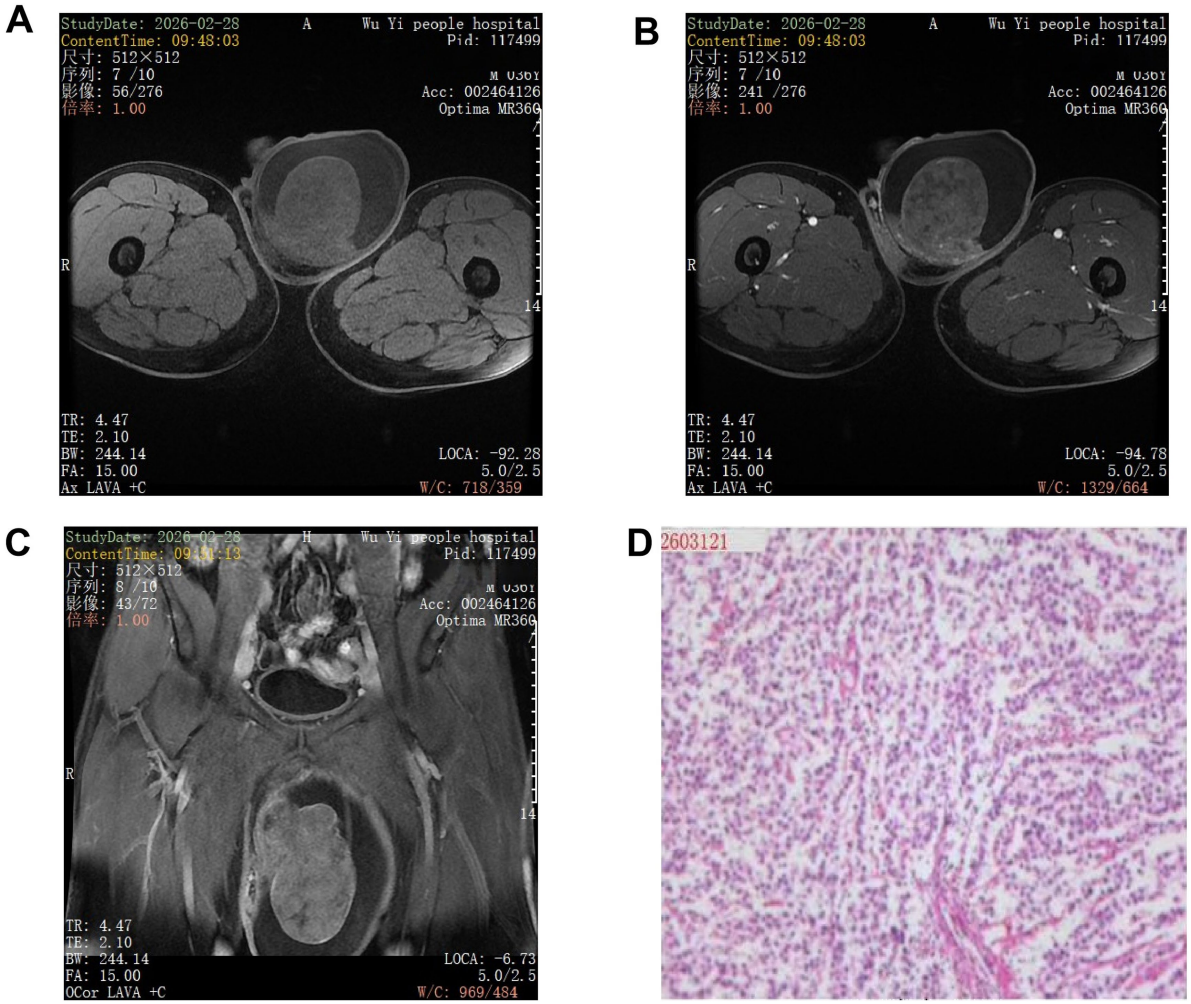
Magnetic resonance imaging of the testicular without contrast agent injection **(A)** and with contrast agent injection (transverse section for **B** and coronal section for **C**), and hematoxylin–eosin staining of the tumor tissue (10 × magnification) **(D)**.

The ultrasound examination revealed that the echo of the left testicle was thickened and uneven, with an unclear boundary from the epididymis (Figure 2). There were slightly abundant blood flow signals. The volume of the left epididymis was enlarged accompanied by thickened and uneven echo. A deep liquid dark area approximately 8.2 cm in depth was observed in the left epididymal sac cavity. The right testicle had a smooth capsule and a smooth surface. Multiple bright spots could be seen in the right testicle, and the internal echo was uniform. The right epididymis was in close contact with the testicle, with normal shape and size, clear and symmetrical boundaries, uniform internal echo, and clear structure. No obvious tortuosity or dilation of the right spermatic vein was observed. Contrast-enhanced CT revealed no abnormalities in the chest, a left hepatic cyst and right hepatic calcification, absence of retroperitoneal lymphadenopathy, and a left scrotal mass suspicious for malignancy (Figure 3). The MRI finding demonstrated a left testicular mass, which was highly suggestive of a seminoma as the primary radiological diagnosis (Figure 4).

### 2.5 Surgical intervention

On March 1, 2026, a radical left orchiectomy with scrotal skin plasty was performed. Intraoperative findings: a 22 × 14 cm irregular, firm mass with poor mobility. The right testis was normal. The specimen weighed 2.51 kg post-resection.

### 2.6 Pathological findings

Gross: The specimen measured 22 × 14 × 12 cm, with attached skin measuring 12 × 9 cm. Sectioning revealed a cystic cavity containing a solid, grayish-white, fish-flesh-like tumor measuring 10 × 7 cm (Figure 4D). Microscopically, the tumor cells are arranged in sheets and nests, separated by fibrous vascular septa, forming a lobular structure. There is uneven lymphocyte infiltration in the interstitium. The tumor cells are large, of uniform size, with large round nuclei in the center, clear nuclear membranes, visible nucleoli, and obvious mitotic figures. The cytoplasm is transparent, acidic, and amphophilic. Large areas of coagulative necrosis are observed, involving the testicular tunica albuginea tissue. Also, there is epididymal tissue, and the resection margin of the spermatic cord is negative. Malignant tumor. The first consideration is spermatocytoma. The tumor cells exhibit an immunohistochemical profile consistent with seminoma (PLAP+, CD117+, SALL4+, D2-40+, Vimentin+, with negative staining for CK(pan), *β*-catenin, Glypican-3, LCA, and CD30), leading to the diagnosis of a malignant tumor, consistent with seminoma.

## 3 Discussion

Giant testicular seminoma is a rare, often slow-growing subtype of testicular germ cell tumor that can reach an exceptionally large size with normal or only mildly elevated tumor markers, and delayed diagnosis, frequently attributable to patient neglect or heavy occupational burdens, remains a key clinical challenge (8, 9). While causal inference cannot be drawn from a single case, the unusual occupational and lifestyle exposures in this patient raise the hypothesis that such factors may warrant further investigation as potential contributors to TGCT development or progression. This case is notable for the tumor’s enormous size (2.51 kg) and the patient’s significant occupational and lifestyle exposures. While cryptorchidism remains the most recognized risk factor for TGCTs (10, 11), emerging evidence suggests that environmental chemicals and lifestyle factors may also play critical roles (12).

### 3.1 Environmental carcinogens and TGCTs

The patient’s prolonged exposure to cooking oil fumes, particularly from high-temperature grilling, would have resulted in exposure to polycyclic aromatic hydrocarbons (PAHs) and other carcinogens. PAHs, such as benzo[a]pyrene, are well-known genotoxic agents that form DNA adducts and induce oxidative stress (6). However, it remains speculative whether this exposure contributed directly to tumorigenesis in this individual patient. Studies have shown that occupational exposure to PAHs increases the risk of various cancers, including testicular cancer (7). Epidemiological studies have also implicated organochlorine pesticides, such as DDE, in the pathogenesis of TGCTs. Cook et al. reported a significant association between serum DDE levels and testicular cancer risk (13). Additionally, Skakkebaek et al. (5) proposed the testicular dysgenesis syndrome (TDS) hypothesis, linking prenatal exposure to endocrine-disrupting chemicals (e.g., phthalates) with increased risk of TGCTs, cryptorchidism, and impaired spermatogenesis.

### 3.2 Lifestyle factors and immune function

The patient’s extreme work schedule (20 h/day, <4 h sleep) likely contributed to chronic sleep deprivation, immunosuppression, and systemic inflammation. Chronic stress and sleep disruption have been shown to impair natural killer (NK) cell activity and alter cytokine profiles (14). Whether such immune dysregulation, if present in this patient, played a role in tumor progression remains unknown, but the possibility warrants further investigation in larger studies. While no direct causal link has been established between sleep deprivation and TGCTs, the cumulative effect of environmental toxins and immune dysfunction may create a permissive environment for malignant transformation. Regarding the observed temporal relationship, a one-year interval between exposure onset and first symptom appears short for *de novo* testicular tumorigenesis. However, testicular germ cell tumors frequently arise from germ cell neoplasia *in situ*, which can remain subclinical for years or decades before invasive growth. Therefore, the occupational exposure in this case may have promoted the progression of a pre-existing subclinical lesion rather than initiated de novo carcinogenesis within 1 year.

In addition to sleep deprivation, the patient’s long-term smoking history (20 cigarettes daily for many years) represents a significant chronic exposure to polycyclic aromatic hydrocarbons and other tobacco-related carcinogens, which may have initiated germ cell neoplasia *in situ* years before the occupational exposure to cooking fumes. Alcohol consumption (1.5 L/day of 3% beer) is not an established independent risk factor for TGCTs, but heavy chronic intake may contribute to systemic immunosuppression and metabolic derangements that could theoretically influence tumor progression. Furthermore, genetic susceptibility cannot be assessed in this single case; inherited variants in genes such as KIT, KRAS, TP53, or those involved in spermatogenesis may modify individual risk. Therefore, smoking, alcohol, and unmeasured genetic factors represent important confounders in interpreting the role of occupational and lifestyle exposures in this patient.

### 3.3 Diagnostic and therapeutic considerations

The patient’s tumor markers were normal, which occurs in approximately 30% of seminoma cases. Imaging, particularly MRI, was instrumental in preoperative diagnosis. Radical orchiectomy remains the standard of care for testicular malignancy, and adjuvant therapy is determined by staging.

### 3.4 Delay in diagnosis

A two-year delay in diagnosis allowed the tumor to reach an extraordinary size. Barriers to timely diagnosis include lack of awareness, socioeconomic factors, and patient neglect. Public health initiatives should emphasize routine testicular self-examination and prompt evaluation of scrotal masses.

### 3.5 Limitations and future directions

This case report has several inherent limitations. First, as a single-case study, it lacks generalizability and cannot establish a causal relationship between environmental exposures and the development of testicular germ cell tumors. Second, although the patient reported prolonged exposure to cooking fumes and severe sleep deprivation, objective quantitative measurements of environmental exposures, such as urinary biomarkers of PAHs (e.g., 1-hydroxypyrene, 2-naphthol) or serum levels of endocrine-disrupting chemicals (e.g., organochlorine pesticides), were not obtained. This limitation, as correctly noted by the reviewer, substantially constrains the strength of the environmental argument and precludes any exposure–response assessment. The absence of such biomonitoring data reflects the constraints of a retrospective case report conducted in a resource-limited primary care setting, where advanced analytical toxicology was not available at the time of patient presentation. Therefore, our environmental hypothesis should be interpreted as hypothesis-generating rather than confirmatory. Third, long-term follow-up data on recurrence, adjuvant therapy, and fertility outcomes are not yet available, given the recent surgical intervention. Testicular germ cell tumors typically arise from germ cell neoplasia *in situ*, which can remain dormant for years or even decades before progressing to invasive cancer. In the present case, the patient reported a two-year history of scrotal enlargement, but the initiation of tumorigenesis likely predated the occupational exposure by an unknown period. His long-term smoking history represents a more chronic exposure to polycyclic aromatic hydrocarbons and other carcinogens. Therefore, we do not propose that the three-year occupational exposure alone caused the seminoma. Instead, we suggest that severe chronic sleep deprivation and sustained exposure to cooking fumes may have acted as promoting or accelerating factors, potentially through mechanisms involving immunosuppression and chronic inflammation, in a patient with pre-existing germ cell neoplasia. This interpretation is consistent with the multifactorial model of TGCT pathogenesis, wherein genetic susceptibility, environmental triggers, and lifestyle factors interact over an extended timeline. While this case report generates a hypothesis linking environmental exposures and extreme lifestyle factors to testicular germ cell tumor development, it does not provide molecular evidence to support a precision medicine approach. Comprehensive molecular profiling including somatic mutation analysis (e.g., KIT, KRAS, TP53), germline susceptibility testing, and immune microenvironment characterization, would be required to establish individualized risk prediction or targeted prevention. Future studies should integrate such molecular data with detailed exposure assessment to move beyond hypothesis generation toward mechanism-based precision strategies. We could not perform germline genetic testing to assess inherited susceptibility, nor can we disentangle the independent contributions of smoking, alcohol, and occupational fume exposure given the single-case design. These confounding factors limit causal inference and underscore the hypothesis-generating nature of this report.

Future research should prioritize large-scale, multicenter epidemiological studies that incorporate detailed environmental exposure assessments and lifestyle evaluations to better elucidate modifiable risk factors for TGCTs. Biomonitoring of carcinogens and endocrine-disrupting chemicals in high-risk occupational groups (e.g., food industry workers, firefighters) could provide valuable insights into early pathogenic mechanisms. Additionally, integrating molecular profiling (e.g., genomic, epigenomic, and immune microenvironment analyses) with comprehensive exposure data, termed the “exposome” approach, may help clarify the complex interplay between environmental insults, immune dysfunction, and testicular carcinogenesis. Prospective cohort studies with long-term follow-up are also warranted to evaluate the impact of lifestyle interventions on reducing TGCT risk and improving prognosis. We recommend that future case–control or cohort studies investigating environmental risk factors for TGCTs incorporate validated exposure biomarkers such as urinary PAH metabolites, serum persistent organic pollutant (POP) levels, or DNA adduct assays alongside detailed occupational and lifestyle questionnaires. In high-risk occupational groups (e.g., barbecue workers, firefighters, chimney sweeps), routine biomonitoring could provide objective evidence linking specific carcinogens to testicular carcinogenesis. Such an “exposome” approach, integrating internal and external exposure measurements, would substantially strengthen causal inference beyond the descriptive level achievable in single case reports.

## 4 Conclusion

This case suggests the hypothesis that chronic environmental and lifestyle factors may influence TGCT progression, but confounding factors (smoking, alcohol, genetics) cannot be ruled out. Causality cannot be established from a single case. Early diagnosis remains critical for optimal outcomes. Clinicians should consider occupational and environmental exposures in the etiological assessment of testicular tumors. Preventive strategies should include reducing exposure to carcinogens, managing occupational stress, ensuring adequate sleep, and promoting regular health screenings. Given the absence of molecular profiling in this case, the current findings should be interpreted as hypothesis-generating rather than as a basis for precision medicine interventions. Larger epidemiological and mechanistic studies are required to test this hypothesis.

## Data Availability

The original contributions presented in the study are included in the article/supplementary material, further inquiries can be directed to the corresponding author.
